# Clinical Management of Pancreatic Cancer

**Published:** 2014-09-01

**Authors:** Rae Brana Reynolds, Justin Folloder

**Affiliations:** University of Texas MD Anderson Cancer Center, Houston, Texas

Pancreatic cancer is the fourth leading cause of cancer deaths in the United States (American Cancer Society [ACS], 2014). In 2014, the ACS estimates 46,420 new cases of pancreatic cancer with 39,590 deaths in the United States. Unfortunately, 80% of patients diagnosed with pancreatic cancer present with metastatic or locoregional disease at initial diagnosis (Chatterjee et al., 2012a; Karmazanovsky, Fedorov, Kubyshkin, & Kotchatkov, 2005). Because metastatic and locally advanced extra-pancreatic disease is an exclusion criterion for surgical treatment, this leaves only a minority of patients initially presenting with pancreatic cancer eligible for surgical resection (Chatterjee et al., 2012a).

The only treatment for pancreatic cancer with curative potential is resection of the involved portion of the pancreas, so with a small subgroup of patients presenting with resectable pancreatic cancer at initial diagnosis, the prognosis for this patient population is grim.

While the 5-year survival rates for many oncologic diseases have improved, the 5-year survival rate for pancreatic cancer remains dismal at 6% (ACS, 2014). Even at high-volume specialty centers, where the 5-year survival rate for patients is higher than in the general population, disease recurrence is still a major problem. For patients who have undergone surgical resection of the involved pancreas, published series from high-volume referral centers examining long-term survivors indicate that only 10% to 27% of patients with early-stage disease who underwent resection survived at least 5 years (Katz et al., 2009).

An MD Anderson Cancer Center (MDACC) analysis of 86 patients who received preoperative radiation and chemotherapy in the form of gemcitabine followed by resection reported that 11% of patients had local pancreatic disease recurrence after resection, 23% had liver metastasis after resection, and 59% had tumor recurrence with distant organ metastasis after resection (Evans et al., 2008).

Another MDACC study of 90 patients who received radiation and chemotherapy in the form of gemcitabine combined with cisplatin reported 25% of study patients presenting with local pancreatic disease recurrence after surgery and 73% of patients had tumor recurrence with distant organ metastasis after surgery (Varadhachary et al., 2008). There is a high frequency of subclinical metastases at initial presentation as well as a high frequency of undetectable extrapancreatic disease at the time of surgical resection, which also contributes to the poor long-term outcomes (Chatterjee et al., 2012b).

## Risk Factors

Although the exact mechanism of cause and effect has yet to be clearly elucidated, tobacco smoking is recognized as a strong risk factor for pancreatic cancer (Lochan, Reeves, Daly, & Charnley, 2011). Other risk factors such as alcohol consumption, chronic pancreatitis, and diabetes mellitus are often mentioned in the literature but require more epidemiologic studies and clinical research for further substantiation. Plasma 25-hydroxyvitamin D, also known as 25(OH)D, has been examined; among participants in five large prospective cohorts, higher plasma levels of 25(OH)D were associated with a lower risk for pancreatic cancer (Wolpin et al., 2012).

Genetics and a family history of disease are recognized risk factors for developing pancreatic cancer as well. Approximately 5% to 10% of patients with pancreatic cancer have a family history of the disease (Hidalgo, 2010). Individuals with the *BRCA2* mutation who are known to have an increased risk for developing breast and ovarian cancers are now recognized to have an increased risk for developing pancreatic adenocarcinoma (Moran et al., 2012). Other genes with variants associated with increased pancreatic cancer risk include *BRCA1, PALB2, ATM, CDKN2A, APC, MLH1, MSH2, MSH6, PMS2, PRSS1, *and* STK11* (Solomon, Das, Brand, & Whitcomb, 2012).

## Presenting Signs and Symptoms

**Figure 1:**
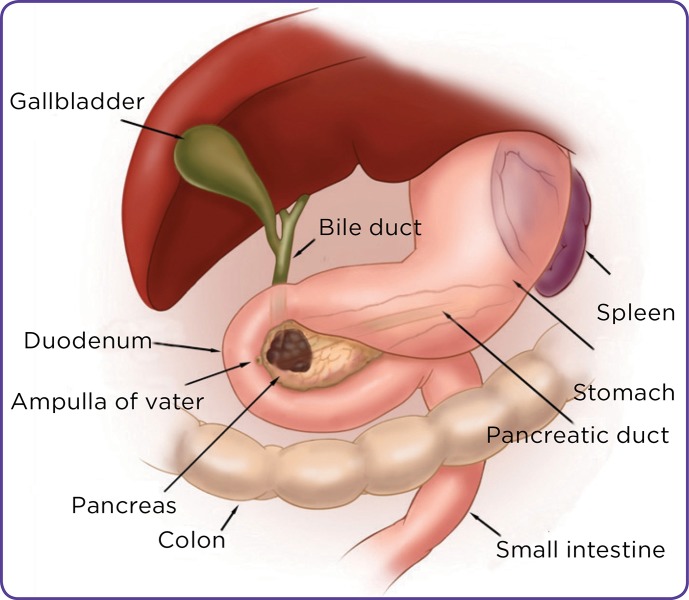
Pancreatic cancer: tumor in the pancreatic head. ©2008 The University of Texas MD Anderson Cancer Center.

Pancreatic cancer will often develop without clear early signs or symptoms, and the eventual manifestations will depend on the tumor location within the gland. Up to 50% of pancreatic cancer patients will present with jaundice, which is more common with patients whose cancers are located in the head of the pancreas where tumors can cause obstruction of the adjacent biliary system (Bose, Katz, & Fleming, 2012). Figure 1 depicts a cancer in the head of the pancreas. Other common manifestations are vague abdominal discomfort, nausea, and weight loss. Large tumors that advance beyond the pancreas can also cause duodenal obstruction or gastrointestinal bleeding. Steatorrhea can also result from obstruction of the pancreatic duct, whereas hyperglycemia and diabetes have been associated with early manifestation of disease. Patients with advanced disease can also present with pain, ascites, and depression. Laboratory study abnormalities can include elevated liver function studies, hyperglycemia, and anemia (Hidalgo, 2010).

## Screening and Early Detection

The relative lack of symptoms at the early stage of disease makes early diagnosis of pancreatic cancer rare. An additional detriment to early diagnosis is the lack of an established standard for screening or prevention, as there is no single reliable test for early detection of pancreatic cancer for the general population (Greenhalf, Grocock, Harcus, & Neoptolemos, 2009). The only screening programs that are currently available are in research settings and are narrowly focused on detecting potentially precancerous lesions among high-risk individuals (Shin & Canto, 2012).

The International Cancer of the Pancreas Screening (CAPS) Consortium summit on the management of patients with increased risk for familial pancreatic cancer reached a consensus that first-degree relatives of patients with pancreatic cancer from a kindred who has at least two affected first-degree relatives and patients with Peutz-Jeghers syndrome are candidates for screening (Canto et al., 2013). The consortium identified mutation carriers of *p16*, *BRCA2*, and hereditary nonpolyposis colorectal cancer with more than one affected first-degree relative as candidates for screening as well. There was no consensus on the age to initiate screening or stop surveillance, but it was agreed that initial screening should include endoscopic ultrasonography (EUS) and/or MRI/magnetic resonance cholangiopancreatography. There was also consensus that surgery, when recommended, should be performed at a high-volume center (Canto et al., 2013).

## Diagnosis

The goals of pancreatic cancer evaluation are to establish a tissue diagnosis of pancreatic cancer and to determine resectability as well as disease stage to help guide treatment planning. In addition to physical examination and a careful history assessment, pancreatic cancer evaluation includes laboratory, diagnostic radiology, and endoscopic studies. Biopsy for cytopathologic tissue diagnosis can be performed with radiology guidance or by endoscopic means (Hidalgo, 2010; Lee & Lee, 2014).

There is no known biomarker specific to pancreatic cancer, but carbohydrate 19-9 (CA 19-9) has demonstrated clinical value for therapeutic monitoring and for surveillance of disease recurrence in patients with a history of pancreatic cancer. It is important to note that CA 19-9 may be elevated during periods of cholestasis and that some patients with pancreatic cancer do not express elevations in CA 19-9, as there is a subgroup of about 10% who are unable to synthesize CA 19-9 and have undetectable levels, even in advanced stages of disease (Hidalgo, 2010).

The diagnostic radiology test of choice for initial pancreatic cancer evaluation is a multiphase, multidetector helical computed tomography (CT scan) with utilization of contrast material (Hidalgo, 2010). This test performed specifically with a pancreatic protocol provides essential details on the anatomic relationship of the tumor to adjacent organs and blood vessels, specifically the superior mesenteric vein, portal vein, superior mesenteric artery, celiac axis, and hepatic artery. A CT scan with contrast can correctly predict resectability in pancreatic cancer with 80% to 90% accuracy (Karmazanovsky et al., 2005). It also provides information on extrapancreatic lesions suspicious for metastatic disease. Positron emission tomography (PET) can be used to supplement CT scan findings during the evaluation and treatment phases.

Endoscopic procedures such as EUS with fine-needle aspiration and endoscopic retrograde cholangiopancreatography (ERCP) are commonly used for pancreatic cancer evaluation (Ross et al., 2008). An esophagogastroduodenoscopy (EGD) with EUS is useful in characterizing tumor details and obtaining tissue diagnosis. It can also be valuable in identifying a cancerous tumor that it is not clearly identifiable on a CT scan as it has better sensitivity for smaller pancreatic lesions (Ross et al., 2008). An ERCP is used for evaluation and management in patients with jaundice and cholestasis. It is used as a diagnostic tool to assess for a biliary stricture resulting from pancreatic cancer obstructing the bile duct and also as a guide in obtaining cytologic brushings of the area of the stricture for cytopathology studies (Hidalgo, 2010). In addition to its value as a diagnostic tool, it is also a therapeutic procedure that guides stent placement to relieve biliary tract compression by pancreatic cancer.

## Staging of Pancreatic Cancer

The tumor-node-metastasis (TNM) classification system issued by the American Joint Committee on Cancer (AJCC) is used to stage pancreatic cancer. The size of the tumor and its relationship to vital blood vessels are taken into account when categorizing the tumor from TX to T4. The extent of regional lymph node involvement defines nodal classification ranging from NX to N1, whereas the presence and/or absence of identifiable metastasis to distant organs designates the metastatic category as M0 or M1, respectively. Table 1 presents details of the levels that comprise each component of the TNM taxonomy, while Table 2 summarizes the AJCC staging system for pancreatic cancer using groupings categorized according to the TNM classification.

**Table 1:**
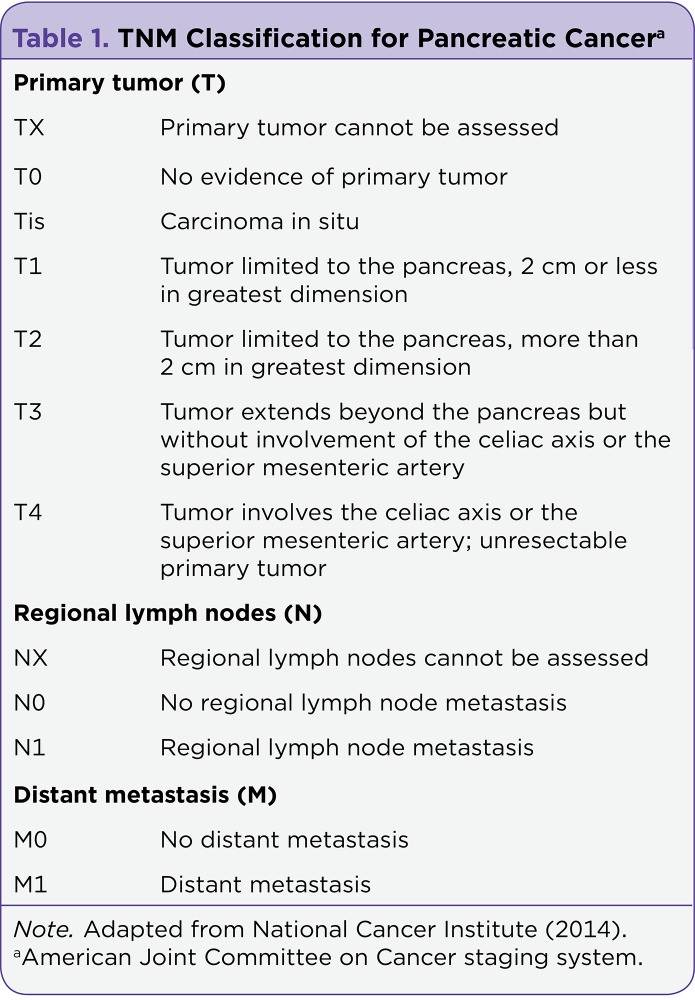
TNM Classification for Pancreatic Cancer

**Table 2:**
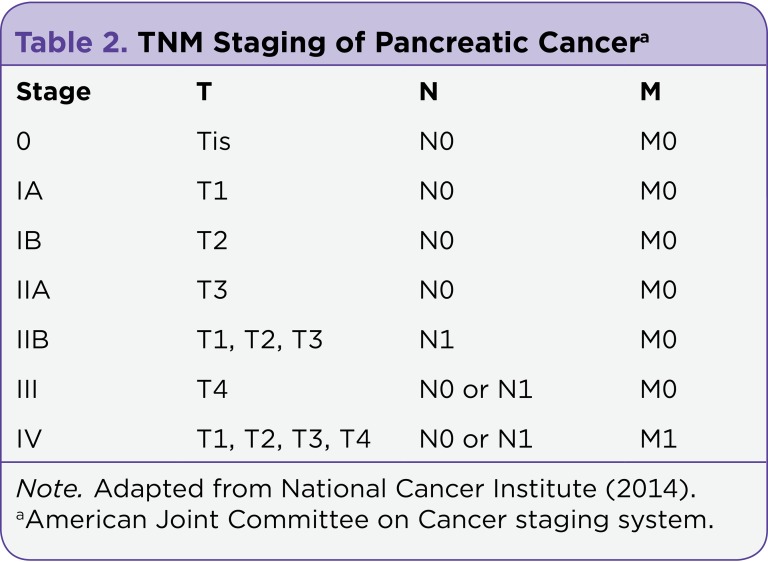
TNM Staging of Pancreatic Cancer

The AJCC staging system has prognostic value but cannot consistently direct clinical management because it requires information that is not always readily available during the initial phase of treatment planning. For example, in most cases, regional lymph node involvement is unknown until after the patient has undergone surgical resection. As such, the primary guide to clinical management of pancreatic cancer at initial diagnosis becomes the patient’s eligibility for surgical resection rather than the TNM staging status. Different clinical staging systems have been developed to categorize pancreatic cancer according to surgical resectability, and these clinical staging systems help steer treatment planning for patients. The MDACC classifies pancreatic cancer as resectable, borderline resectable, locally advanced, and metastatic.

*Resectable disease* is characterized by the absence of extrapancreatic disease; a patent superior mesenteric vein-portal vein (SMV-PV) confluence; and clear tissue planes between the celiac axis (CA), superior mesenteric artery (SMA), and the common hepatic artery (Bose et al., 2012). At our institution, MDACC, *borderline resectable disease* is characterized by the absence of extrapancreatic disease and the presence of tumor involvement or occlusion of the SMV-PV confluence that is amenable to resection and reconstruction, tumor abutment of the SMA for less than 180º of its circumference, and short segment encasement of the hepatic artery (Bose et al., 2012).

**Figure 2:**
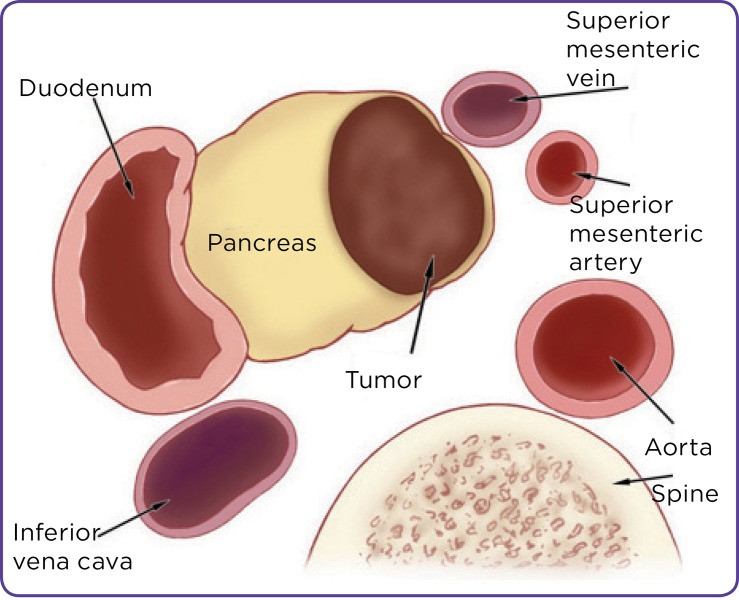
Resectable pancreatic cancer. ©2008 The University of Texas MD Anderson Cancer Center.

**Figure 3:**
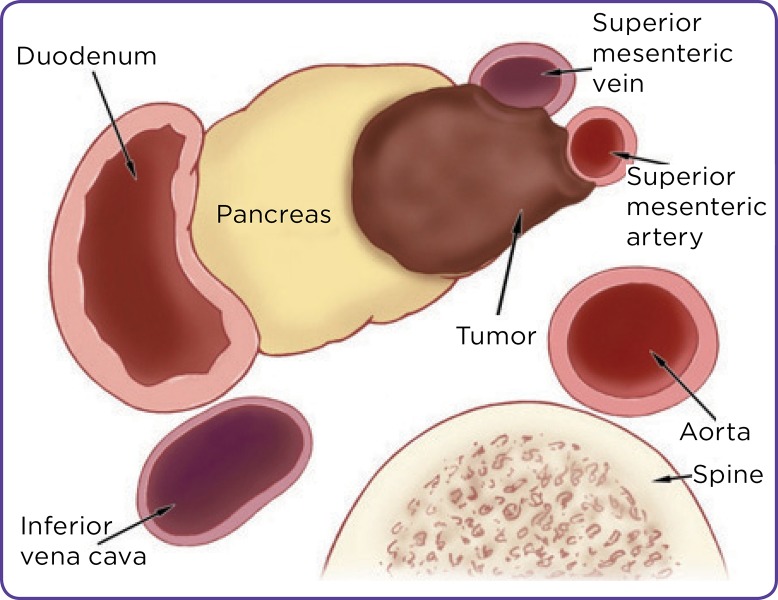
Borderline resectable pancreatic cancer. ©2008 The University of Texas MD Anderson Cancer Center.

**Figure 4:**
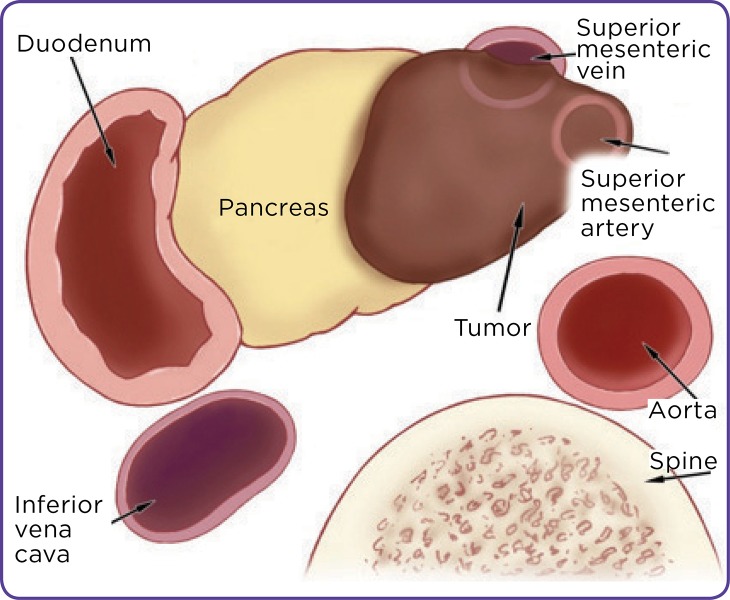
Locally advanced pancreatic cancer. ©2008 The University of Texas MD Anderson Cancer Center.

*Locally advanced disease* is characterized by the presence of tumor encasement of the SMA or CA for greater than 180º of its circumference in the absence of extrapancreatic disease (Bose et al., 2012). Table 3 summarizes the criteria for these three clinical staging categories used for determination of resectability, and Figures 2, 3, and 4 provide illustrations of the different categories. In addition to these three, there is the category of *metastatic disease,* which is characterized by radiographic or clinical evidence of pancreatic cancer that has spread to distant organs or the peritoneum.

**Table 3:**
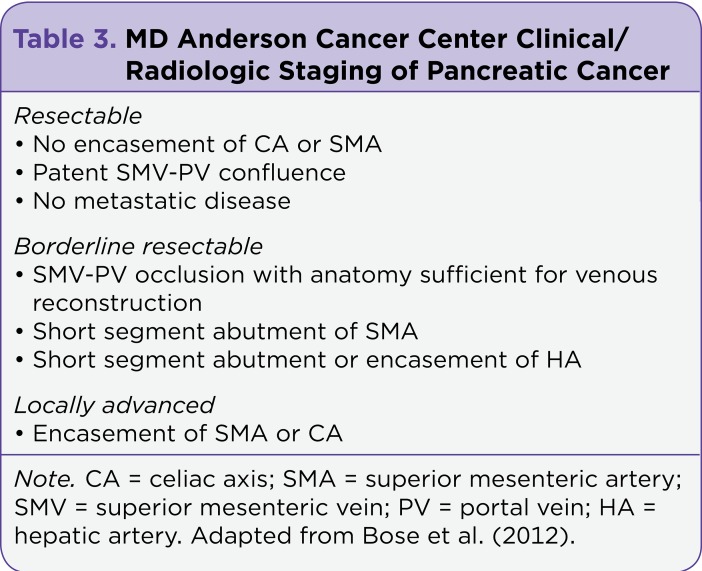
MD Anderson Cancer Center Clinical/ Radiologic Staging of Pancreatic Cancer

## Treatment

Treatment and clinical management of pancreatic cancer are often determined by the clinical stage of the patient’s disease and are usually focused on the question of disease resectability. Patients who have resectable disease are eligible for surgery and thus have a significantly improved prospect for long-term survival and cure. Chemotherapy, radiation, and surgery are utilized in the treatment of pancreatic cancer; the modalities utilized as well as the sequence in which they are administered often depend on the clinical stage of disease.

**Resectable Pancreatic Cancer**

For resectable pancreatic cancer, the primary recommendation from the National Comprehensive Cancer Network (NCCN) is to proceed immediately to surgical resection followed by adjuvant chemotherapy. However, there is also expert consensus and phase II clinical trial data that support the delivery of neoadjuvant therapy (i.e., chemotherapy and radiation administered prior to surgical resection) in selected patients with biopsy confirmation of adenocarcinoma (Halperin & Varadhachary, 2014).

The primary chemotherapeutic agents that have shown benefit in patients with pancreatic cancer are gemcitabine and fluorouracil (5-FU). The use of gemcitabine has shown an increased median disease-free survival to 13.4 months compared with 6.7 months in an observation group (Oettle et al., 2007). A 5-year actuarial survival of 21% was seen in patients treated with adjuvant 5-FU compared with 9% in patients randomized to receive nonadjuvant treatment (Halperin & Varadhachary, 2014).

There is conflicting information on the role of radiation in patients with resectable pancreatic cancer. A 2004 study confirmed that patients may benefit from adjuvant chemotherapy but showed a lower survival rate among patients treated with adjuvant chemotherapy combined with radiation when compared with patients who did not receive adjuvant chemotherapy and radiation (Neoptolemos et al., 2004). These results are in contrast to findings of multiple trials that have suggested a survival benefit in pancreatic cancer patients treated with radiation (Corsini et al., 2008; Herman et al., 2008; Hsu et al., 2010).

For patients with resectable pancreatic cancer, the delivery of neoadjuvant therapy is advocated by some centers, as it allows for early treatment of systemic disease in a population of patients widely believed to have micrometastasis at presentation (Evans et al., 2008). Additionally, it allows for identification of patients with rapidly metastatic disease and spares them from major operation unlikely to provide durable cure because of highly aggressive tumor biology. Moreover, delivery of neoadjuvant chemotherapy and radiation is posited to increase the rate of margin-negative resection (R0 resection) and reduce the risk of local disease recurrence (Evans et al., 2008).

Achieving microscopically negative surgical margins of resection is the goal of any pancreatic cancer operation (Evans et al., 2009). Surgical resection of pancreatic cancer is performed for patients whose radiographic imaging studies indicate resectable disease and whose clinical performance status is appropriate for surgery. The majority of pancreatic cancers arise in the area of the pancreatic head, and these lesions, if resectable, are treated with a pancreaticoduodenectomy. Pancreatic cancers located in the area of the pancreatic tail, if resectable, are treated with a distal pancreatectomy, which typically also involves a splenectomy depending on the splenic vessel involvement of the tumor.

**Figure 5:**
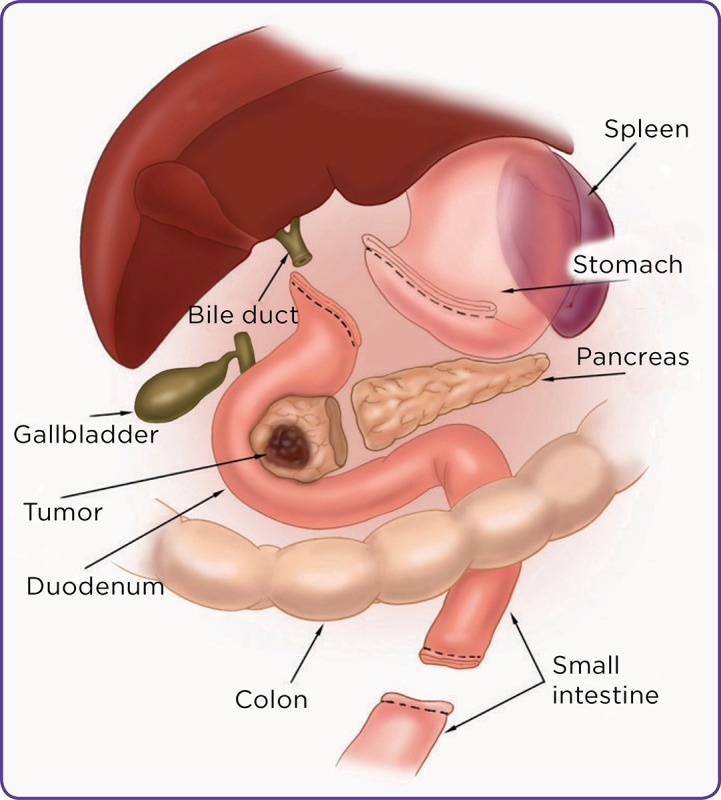
Pancreaticoduodenectomy: resection of distal stomach, bile duct, gallbladder, duodenum, head of the pancreas. ©2008 The University of Texas MD Anderson Cancer Center.

**Figure 6:**
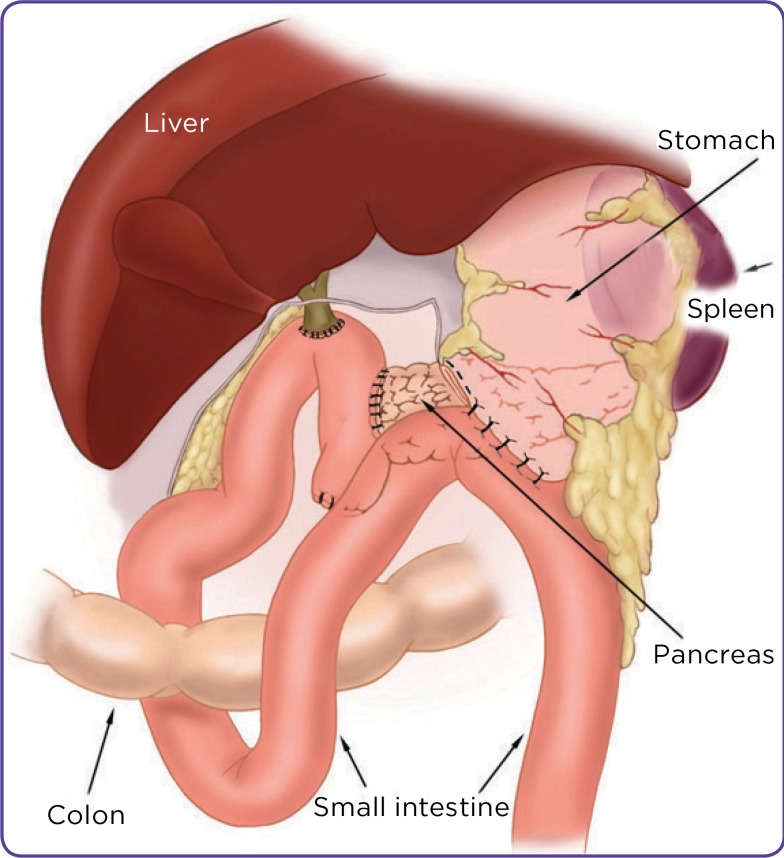
Pancreaticoduodenectomy: postresection anastomoses. ©2008 The University of Texas MD Anderson Cancer Center.

A pancreaticoduodenectomy is commonly referred to as the Whipple procedure and involves the resection of the pancreatic head, duodenum, gallbladder, and a portion of the stomach (Figure 5). Reconstruction is then performed with a pancreaticojejunostomy, choledochojejunostomy, and duodenojejunostomy or gastrojejunostomy (Figure 6). A pancreaticoduodenectomy for resectable pancreatic cancer can be performed with pylorus preservation, especially in patients with a high risk for postoperative nutritional compromise. However, it is not typically performed in patients with bulky pancreatic head tumors that involve adjacent organs or in the presence of grossly positive pyloric or peripyloric lymph nodes (Bose et al., 2012).

Involvement of the SMV-PV is not considered an absolute contraindication to resection of pancreatic cancers at our institution. In 84% of patients, imaging studies can accurately predict the need for resection and reconstruction of the SMV-PV due to tumor involvement (Bose et al., 2012). Resection and reconstruction of the SMV-PV have been found to be safe. It has also been found that patients who require resection and reconstruction of the SMV-PV to achieve negative surgical margins have a similar overall survival rate as patients who undergo resection without the need for venous resection and reconstruction (Bose et al., 2012). At our institution, most patients with resectable pancreatic cancers who have venous involvement are treated preoperatively with chemotherapy and radiation. Expert consensus states that pancreaticoduodenectomy for pancreatic adenocarcinoma should be performed at high-volume institutions capable of and experienced in resection and reconstruction of major mesenteric veins (Evans et al., 2009).

**Borderline Resectable Pancreatic Cancer**

Because this category is relatively new and does not yet have a standard definition among institutions and organizations with expertise in pancreatic cancer management, standard treatment for borderline resectable disease is not well established; however, expert consensus statements were published in 2009 on the surgical treatment and combined-modality treatment of resectable and borderline resectable pancreatic cancers (Abrams et al., 2009; Evans et al., 2009)

Heterogeneity in the definitions as well as the interventions used by different institutions that have conducted studies on patients with borderline resectable disease remains. Multiple trials support the delivery of preoperative neoadjuvant therapy either with chemotherapy alone or in combination with radiation, including the use of FOLFIRINOX, which is a combination of 5-FU, oxaliplatin, irinotecan, and leucovorin followed by chemoradiation (Christians et al., 2014). However, there is no consensus standard on which chemotherapy or chemoradiation regimen in particular should be delivered prior to resection (Halperin & Varadhachary, 2014).

**Unresectable Pancreatic Cancer**

Locally advanced and metastatic pancreatic cancers are considered unresectable. Because resection of the involved pancreas is the only treatment that offers cure, unresectable disease is therefore considered incurable. In these palliative settings, there is no role for resection, and treatment usually consists of systemic chemotherapy and in some cases with chemoradiation. The role of radiation in combination with chemotherapy for locally advanced disease has been studied: Although chemoradiation has shown benefit, there has also been a noted increase in toxicity. The added morbidity and the low enrollment in clinical trials designed to examine its benefit have precluded a firm conclusion on its status as recommended treatment (Hidalgo, 2010).

For systemic chemotherapy agents, gemcitabine has long been considered the treatment of choice for unresectable and metastatic pancreatic cancers. It has been used as a single agent and also in combination with other agents for treatment of advanced disease (Hidalgo, 2010). In a study published in 2011, FOLFIRINOX was shown to offer a survival advantage but increased toxicity compared with gemcitabine in patients with advanced disease (Conroy et al., 2011). As such, FOLFIRINOX has become an option for patients with metastatic disease, provided that they have an otherwise good performance status.

One randomized phase III study found that weekly gemcitabine combined with albumin-bound paclitaxel had a statistically significant prolongation of median overall survival in patients with metastatic pancreatic cancer when compared with single-agent gemcitabine (Von Hoff et al., 2013). This multinational, multi-institutional study of 861 patients yielded a median overall survival of 8.5 months for patients treated with albumin-bound paclitaxel combined with gemcitabine compared with 6.7 months for patients treated with gemcitabine alone. The patient group treated with combination therapy had a 1-year survival rate of 35% compared with 22% for the group that received single-agent gemcitabine; the 2-year survival rate was 9% for the group that received combination therapy and 4% for the group that received gemcitabine alone (Von Hoff et al., 2013).

## Surveillance

At the MDACC, patients undergo posttreatment surveillance every 4 months for the first 2 years following completion of pancreatic cancer treatment; the visits are spread out to every 6 months after 2 years (Bose et al., 2012). The visits consist of a physical examination and surveillance tests including CT scan, serum CA 19-9, and chest x-ray. After 5 years, surveillance visits are scheduled on an annual basis (Bose et al., 2012). 

## Future Directions

Patients with pancreatic cancer encounter challenges throughout the different phases of their illness. Although some advances have been made in the evaluation and treatment of these patients, the poor prognosis associated with this disease underscores the need for continued efforts to enhance understanding of the underlying disease biology to promote progress in finding effective treatments.
